# Impact Analysis of Potential Induced Degradation on Crystalline Silicon Solar Cell Performance by Correlating Practical Diagnosis with MATLAB Simulation

**DOI:** 10.3390/ma15228056

**Published:** 2022-11-15

**Authors:** Abdulwahab A. Q. Hasan, Ammar Ahmed Alkahtani, Mohammad Aminul Islam, Yazan A. Alsariera, Santhiran Sathiswary, Nabilah M. Kassim, Mohammad Ismail Hossain, Yasuaki Ishikawa, Nowshad Amin

**Affiliations:** 1 Institute of Sustainable Energy, Universiti Tenaga Nasional, Jalan IKRAM-UNITEN, Kajang 43000, Selangor, Malaysia; 2 Department of Electrical Engineering, Faculty of Engineering, University of Malaya, Jalan Universiti, Kuala Lumpur 50603, Selangor, Malaysia; 3 Department of Computer Science, College of Science, Northern Border University, Arar 91431, Saudi Arabia; 4 Department of Electrical and Computer Engineering, University of California, Davis, CA 95616, USA; 5 Marvell Nanofabrication Laboratory (Nanolab), University of California, Berkeley, CA 94720, USA; 6 College of Science and Engineering, Aoyama Gakuin University, 5-10-1 Fuchinobe, Sagamihara 252-5258, Kanagawa, Japan

**Keywords:** degradation, defects, fill factor, EL, energy, MATLAB/Simulink, parasitic resistance, potential induced degradation, solar cell performance

## Abstract

Extensive research on fault diagnosis is essential to detect various faults that occur to different photovoltaic (PV) panels to keep PV systems operating at peak performance. Here, we present an impact analysis of potential induced degradation (PID) on the current–voltage (I-V) characteristics of crystalline silicon (c-Si) solar cells. The impact of parasitic resistances on solar cell performance is highlighted and linked to fault and degradation. Furthermore, a Simulink model for a single solar cell is proposed and used to estimate the I-V characteristics of a PID-affected PV cell based on experimental results attributes. The measured data show that the fill factor (FF) drops by approximately 13.7% from its initial value due to a decrease in shunt resistance (R_sh_). Similarly, the simulation results find that the fill factor degraded by approximately 12% from its initial value. The slight increase in measured data could be due to series resistance effects which were assumed to be zero in the simulated data. This study links simulation and experimental work to confirm the I-V curve behavior of PID-affected PV cells, which could help to improve fault diagnosis methods.

## 1. Introduction

Solar photovoltaic systems (PVs) are expected to account for 710 GW of global cumulative installations by the end of 2020, up from 635 GW in 2019 [1]. With such rapid growth, the need for dependable monitoring and fault diagnosis systems has become a real necessity to maintain PV system performance at peak output levels. PV systems are subjected to numerous failures and degradations during their operational years, which are caused by either ambient conditions [2] or system configuration error [3]. Certain types of these faults go undetected for a portion of operational time, resulting in output power loss. As a result, it is critical to understand fault behavior in greater detail to develop a more realistic fault diagnosis system.

Visual inspection technique is utilized to identify PV module and system faults, such as discoloration, browning, junction box failure, and delamination. Electroluminescence (EL) and thermal imaging are also used to detect hidden cracks and hotspots [4]. Electrical inspection, such as illuminated/dark curve measurement, is another technique to identify faults in PV modules and systems. Using electrical signatures to detect failures is more advantageous and promising for diagnosing and monitoring PV systems [5,6]. Various types of PV module faults were studied and analyzed using simulation work [7,8,9]. These faults are specified for PV array systems, such as open-circuit, short-circuit, connection, and shading faults, as well as aging faults to some extent. W. Chine et al. [10] developed a novel fault diagnosis technique based on I-V characteristics to identify eight types of PV array faults. The author compared simulation and measurement data of four series-connected PV modules’ I-V characteristics. The results showed that the simulated and measured data agreed well, with minor error ranges of 1.1–1.6%, 1.5–4.7%, 1.5–4.7%, and 1.1–3.8% for short circuit current (I_sc_), maximum output current, open-circuit voltage (V_oc_), and maximum output voltage, respectively.

Degradation faults are considered more difficult to define because they are related to several factors and influence several electrical parameters, resulting in a serious malfunction in the PV system or module. This refers to the deterioration of PV module components/materials (glass, encapsulation, seals, etc.). For example, electromigration in metal fingers of solar cells may cause an increase in the temperature of affected areas. As a result, encapsulant material may deteriorate, causing delamination failure [11]. In addition, potential induced degradation (PID) is one of the degradations that reduces output power by more than 40% in less than a year due to the high leakage current caused by a high voltage system [12]. Because there are no visible symptoms of PID defect in PV modules, it is more difficult to detect it in the early stages and is detected only when degradation is severe. Recently, researchers have begun to implement methods to detect PID failure in PV systems at earlier stages [13]. PID is typically detected using various methods, such as measuring shunt resistance [13,14], thermal or EL imaging [15,16], and measuring open-circuit voltage at both ends of a PV string in low light conditions [17]. The mode of PID failure is loss of fill factor which is associated with decreased shunt resistance when measuring the IV curve and indicates PN junction deterioration by creating a parallel recombination path within the cell [18]. SV Spataru et al. [19] proposed a fault identification method based on light and dark IV characteristics to distinguish four types of degradation modes, including PID. On the basis of light IV data at standard test conditions, they discovered that the common losses associated with PID fault are maximum output current and fill factor (FF).

Our research will contribute to a better understanding of the I-V curve behavior under fault conditions at the cell level and will aid in estimating the I-V curve behavior at module and array levels. As previously stated, the majority of simulation work focuses on general PV system faults such as short-circuiting or shading. Furthermore, there is a lack of explanation about the root cause of degradations and parameters affected by specific faults, resulting in only a high-level view of output power. As a result, the PID fault will be investigated in this study. We analyzed the solar cell using real experimental work and compared the results with MATLAB/Simulink model results. The comparison was based on localized affected regions of the solar cell area to predict the performance deterioration of a solar cell due to the presence of a PID fault.

## 2. Solar Cell Characteristics and Parasitic Resistance

**Figure 1:**
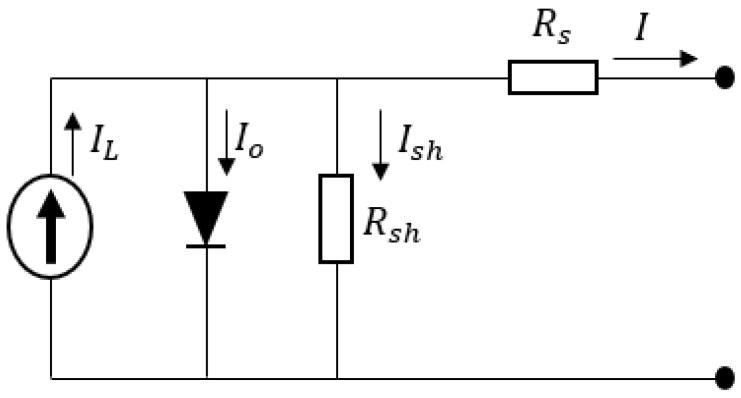
One-diode model of Solar Cell.

The one-diode and double-diode models are commonly used to describe the electrical properties of solar cells. However, the one-diode model is the one more commonly used for greater accuracy and simplicity. Figure 1 depicts the main elements of this model, with the corresponding characteristic equation represented by Equation (1).

(1) $$I=I_{L}-I_{o}\left[exp\left(\frac{V+IR_{s}}{nV_{t}}\right)-1\right]-\frac{V+IR_{s}}{R_{sh}}$$

where *I_L_* stands for photogenerated current; *I_o_* stands for diode saturation current; *R_sh_* stands for shunt resistance; *Rs* stands for series resistance, diode ideality factor *n*; and *Vt* stands for thermal voltage (KT/e), where T is the temperature, e is the electron charge, and k is the Boltzmann constant. These parameters represent the solar cell’s operation state, and monitoring them provides information about electrical and mechanical mismatches. As a result, for a healthy PV system, these parameters remain stable; however, any changes in their values may indicate temporary failure or long-term degradation [20]. From the standpoint of PV module failures and degradation, we will demonstrate the effects of series and shunt resistances on solar cell performance, as well as the corresponding degradation modes that are responsible for resistance value changes.

### 2.1. Shunt Resistance

Shunts cause leakage current and are the local alternative paths of short-circuit current in the solar cell, which is depicted as parallel resistance in the solar cell equivalent circuit model [21,22,23]. Fill factor and open-circuit voltage are generally affected when shunt resistance is reduced, resulting in a reduction in the overall maximum power of the solar cell [24]. At the same time, the reliability of the cell itself or the module is affected due to the heating caused by local shunting. Therefore, heating caused by local shunting compromises the reliability of the cell or module. Shunt resistance can be reduced for a variety of reasons, including impurities near the PN junction that can form during the manufacturing process. Poor edge isolation, crystal defects [25], and solar cell cracks [4] are examples of these, as they increase leakage current and thus shunt resistance. PID failure is another factor that contributes to shunt resistance losses.

PID is considered major reliability trouble for PV modules. This type of degradation happens in PV strings when PV modules are connected in series, which creates voltage potential in the string. As a result, the PV modules will exhibit a potential difference of 1000 V to 1500 V between the cells and the frame [26]. As a result of this electric field, sodium ions (Na+) contained in the glass start to migrate to the solar cell through the encapsulant layer [12]. These Na+ create a leakage current upon reaching the cell’s surface and deteriorate the PV module performance due to the reduced shunt resistance [27].

### 2.2. Series Resistance

In the design of a solar cell, the series resistance is a combination of resistances from different layers. It is made up of four components: material bulk resistance, emitter resistance, contact resistance, and metal resistance. Each of these areas can contribute to the overall losses of series resistance. Manufacturing flaws that cause increased series resistance are frequently inhomogeneously distributed across the solar cell plane. Imperfect contact formation or problems during screen printing, for example, could arise during the firing process [28]. Furthermore, the type and technology of metal used in the metallization process of a solar cell may influence the series resistance value. According to Bahabry RR et al., using NiSi/Cu metallization for rear contact of the c-Si solar cell reduces the value of series resistance by 33.1% when compared with Ag–Al screen printed [29]. A summary of the common defects that affect series resistance is provided in [30] for c-Si PV modules. These include broken interconnects, metallization corrosion, delamination, and failures of solder bonds.

## 3. Methodology

### 3.1. Experimental and Characterization Procedure

A PID failure experiment was performed to investigate the shunting defects on a 156 mm × 156 mm commercial p-type c-Si solar cell to validate the simulation model proposed in this paper. The sample structure is identical to the standard PV module which is made up of two sets of 200 mm × 200 mm soda–lime glass, two sets of 0.43 mm thick encapsulation ethylene-vinyl acetate (EVA) films, and a back-sheet. It is critical to note that the PV module sample was not laminated during the experiment to avoid any difficulties when extracting the solar cell after the PID stress test was completed.

**Figure 2:**
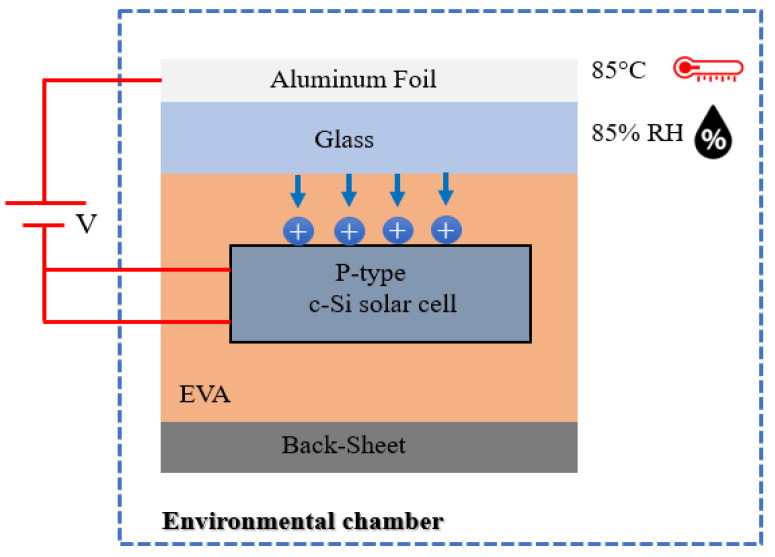
Schematic of PID test setup.

The PID stress test was carried out following the International Electrotechnical Commission (IEC) IEC 62804-1 standard test procedures. To ensure uniform distribution of the voltage applied to the surface of the PV module sample, an aluminum plate with conductive rubber was placed on top of the glass and connected to the power supply’s positive terminal. The PV module’s positive and negative terminals were short-circuited and connected to the power supply’s negative terminal, as shown in Figure 2. PID was provoked by biasing of “−1000 V” concerning the modules glass frame in a climate chamber at environmental conditions (85 °C/85% of RH) and a duration of 100 h.

Sun-illuminated I-V measurement, electroluminescence imaging, and lock-in-thermal (LIT) imaging were used to characterize the test sample before and after the PID stress test. The solar cell’s electrical parameters, such as power, open-circuit voltage, short-circuit current, fill factor, series resistance, and shunt resistance, were measured from the one sun-illuminated I-V under the AM1.5 G condition. Moreover, the EL image was captured in forwarding bias with a current of 40$\;\mathrm{mA}/{\mathrm{cm}}^{2}$ using the InGaAs camera (model: Xeva 1.7 320) at the resolution of 320 × 256 pixels with a 30 µm pixel pitch. LIT images were captured by an InSb camera, cooled at approximately 200 K using a THEMOS-1100L system (Hamamatsu Photonics K.K., Shizuoka, Japan).

### 3.2. Simulation Modeling

**Figure 3:**
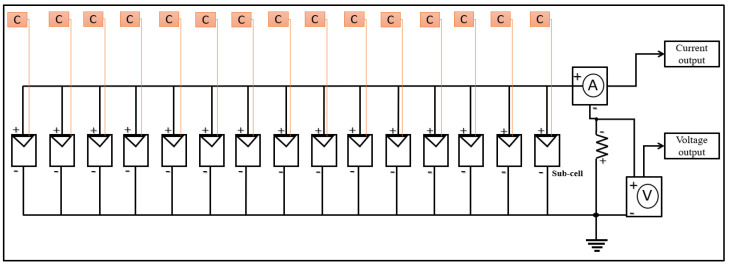
Solar Cell Simulink Model.

This work’s solar cell modeling is based on a single solar cell divided into 15 sub-cells that are connected in parallel. As shown in Figure 3, each sub-cell represents a portion of the entire solar cell and is connected to an individual irradiance source block. A clear image of the Simulink diagram can be seen in Supplementary Materials Figure S1. When connected in series or parallel, solar cells obey Kirchhoff’s voltage and current laws. In a series connection, the output voltage is added together while the current remains constant; in a parallel connection, the current is added together while the voltage remains constant [31]. It should be noted that the short-circuit current for a high-quality industrial c-Si solar cell with a surface area of 156 mm^2^ is approximately 10 A. Details of the Simulink setup and the code for drawing the I-V curve are described in Supplementary Materials with attached Figures S2–S4.

This paper’s simulation model focuses on the PID fault of solar cells caused by shunting defects. On the basis of cell area, assumptions have been made about shunting defects that cause the decrease in shunt resistance and how they affect the I-V curve behavior. This was accomplished by changing the parameter values for a group of sub-cells at various positions. Nonetheless, whether the sub-cell is located at the beginning, middle, or end of the connection, the effects have a homogeneous distribution. As a result, the first three sub-cells were selected as defective regions. Furthermore, simulation model assumptions were based on real experimental results related to the PID defect, which will be analyzed and correlated with the simulation modeling results.

## 4. Results and Discussions

**Figure 4:**
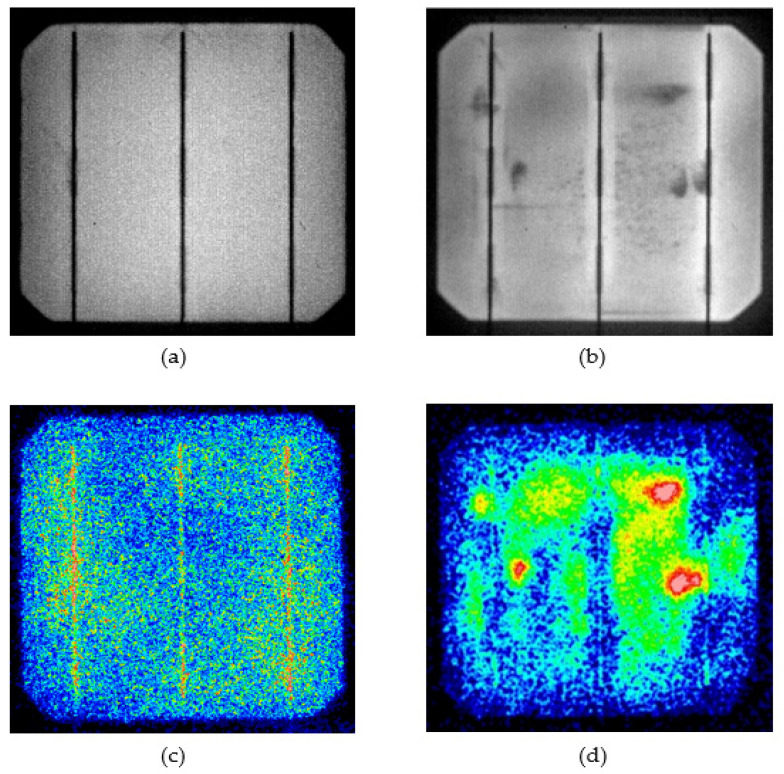
(**a**,**b**) are EL, and (**c**,**d**) are LIT images of single-cell PV modules at fresh and after PID stress test.

Figure 4 shows EL and LIT images for a fresh solar cell sample after 100 h of PID stress testing. The influence of PID stress resulted in three major shunting regions. Furthermore, EL images showed that these regions appeared dark throughout the PID stress test duration. These areas were also visible as hot spots in LIT images, which corresponded to shunting regions in EL images. It has been stated that the initiation of this hot spot is due to the defective regions’ lower short-circuit current compared with the overall current of the solar cell area, causing them to be in reverse biasing. As a result, any generated power will be dissipated as heat energy, indicating performance degradation. The dark regions of the EL images and the hot regions of the LIT images in the affected locations are proof of PID by junction leakage caused by a 93% decrease in shunt resistance from the initial value.

**Figure 5:**
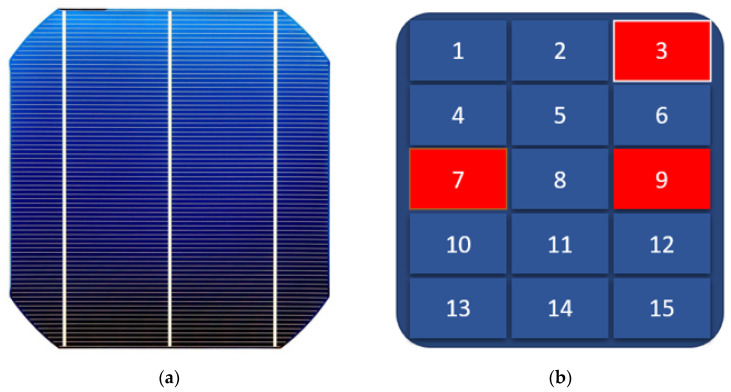
(**a**) Commercial Single c-Si Solar Cell, (**b**) Single Solar Cell with Assumed Defect Areas.

To compare experimental results with Simulink model results, the assumption was made that only three regions of the cell are affected by shunt resistance reduction. This assumption is based on LIT and EL images of experimental results of PID stress, as shown in Figure 4, which shows several hot areas and dark areas in the EL image. Figure 5 depicts a solar cell with presumed defect areas. As mentioned in the previous section, many defects in the manufacturing phase or during field operation can affect series and shunt resistance.

**Figure 6:**
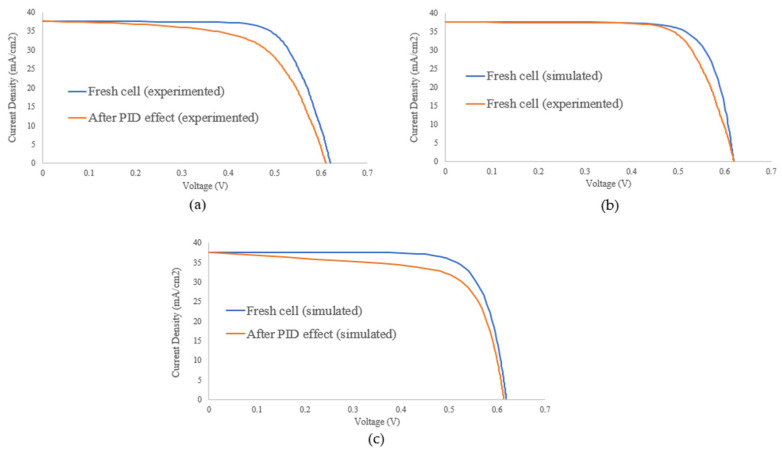
Validation of the Simulation model: (**a**) One sun-illuminated I-V curve fresh and after PID stress test, (**b**) I-V curves of simulated and measured fresh cells, and (**c**) IV curve of simulated PID effect.

Before and after the PID test, the electrical characteristics of the solar cell were measured. Figure 6a depicts the characteristics of one sun-illuminated I-V curve which shows significant power degradation due to the evaluation of PID effects. Table 1 depicts electrical properties such as efficiency (η), fill factor, open-circuit voltage, short-circuit current, and shunt resistance. The open-circuit voltage decreases slightly while the short-circuit current remains constant. After PID stress, shunt resistance decreased significantly from 25.41 Ω to 1.435 Ω, which could be attributed to the presence of sodium ions which are responsible for increased non-radiative carrier recombination and increase in leakage current paths. The efficiency of the solar cell sample decreased by 14.7%, indicating that PID has a significant impact on cell performance. Furthermore, the fill factor dropped dramatically from 73.6% to 63.46%, owing to the decrease in shunt resistance. This is consistent with the findings of [32,33] who stated that the mode of PID failure is a loss of fill factor which is related to decreased shunt resistance when measuring the IV curve.

The simulated and measured IV curves of fresh solar cells are shown in Figure 6b. The curve of measured data is steeper than the curve of simulated data at high voltage ranges, owing to series resistance effects. The logical explanation for this phenomenon is that manufactured solar cells contain natural series resistance which is impossible to avoid, as explained in Section 2; however, the series resistance was assumed to be zero in the simulation graph.

Figure 6c depicts the effect of simulated PID on solar cell performance. In Simulink modeling, the solar cell is considered to have infinite shunt resistance; thereby, only the first three cells model were modified to have a shunt resistance of 1.5 Ω, 1.5 Ω, and 1.8 Ω, respectively, while the remaining cell models were treated as unaffected regions. The simulation result follows a similar pattern to the experimental PID stress. Table 1 shows the electrical parameters of the solar cell model as a result of the reduced shunt resistance. The effect of reduced shunt resistance cannot be overlooked as only three regions are affected, resulting in a power loss of up to 13% of the output power.

Furthermore, there is no discernible difference in open-circuit voltage or short-circuit current. However, the fill factor was the most affected parameter, with a 12% reduction from the initial value due to the effect of shunt resistance, which exhibits almost identical behavior to PID stress results. The small difference between the simulated and measured fill factor is due to the series resistance after PID stress, as shown by the I-V curve. Because of the complexity of this phenomenon, the series resistance in the simulation model was ignored.

For comparing the simulated and the experimented results, the percent of error was calculated using the following equation.

(2) $$\%error=\left[\frac{\left|Simulated\;values-Experimented\;values\right|}{Experimented\;values}\right]\times 100$$

The percentage of errors is shown using “Δ” in Table 1. Overall, the simulated and experimented results show a very low error, and in some cases, it is zero. ΔVmp and ΔFF of simulated and experimented values after the PID effects show 7.9% and 8%, respectively. These values are the highest percentage error among others. The simulation outcomes represent the behavior patterns of the same actual solar cell based on its mathematical model in Equation (1). The Simulink solved the model equation, then processed the equation results based on the input values to obtain the desired performance parameters. Nonetheless, the percentage errors occurred as a result of the discrepancy between the real and estimated cell designs.

## 5. Conclusions

In conclusion, this study has added useful information to our understanding of the I-V curve behavior under fault conditions at the cell level, which helps to predict the I-V curve behavior for module and array levels. Impact analysis of PID fault on I-V characteristics has been discussed by linking experimental work and a simulation model using MATLAB/Simulink for a c-Si solar cell. Furthermore, the impact of parasitic resistances was discussed, particularly series resistance, which is more responsible than shunt resistance. The influence of the PID stress test was identified and located using EL and LIT characterizations which revealed three shunting regions at different locations. Experiential results revealed that shunt resistance was reduced by more than 90% from the initial value, causing the fill factor to drop by more than 13% from the initial value. Furthermore, the I-V curve of the Simulink modeled solar cell successfully matched the real solar cell with little variation in the curve at high voltage. The simulation results based on the shunt resistance effect were found to be in good agreement with the measured results having a small error of 1.78%. The findings of this study may help in the improvement of the diagnostic system for solar PV system failures. In future work, the PID behavior could be investigated on the I-V curve of a p-type solar cell under positive potential voltage. In addition, the proposed single solar cell model could be improved to investigate other degradations that have complex behavior, such as corrosion or metallization degradations.

## Figures and Tables

**Table 1:** Electrical Characteristics of Experimented and Simulated Before and After PID Stress.

Electrical Characteristics	Isc (A)	ΔIsc (%)	Voc (V)	ΔVoc (%)	Imp (A)	ΔImp (%)	Vmp (V)	ΔVmp (%)	FF (%)	ΔFF (%)	ƞ (%)	Δƞ (%)
Fresh	9.138	0	0.6201	0.02	8.525	0.3	0.4892	6.3	73.6	6	17.14	6.2
After PID	9.163	0.3	0.61	0.7	7.675	0.3	0.4634	7.9	63.64	8	14.62	8
Fresh (simulated)	9.138	0	0.62	0.02	8.5	0.3	0.52	6.3	78	6	18.2	6.2
After PID (simulated)	9.138	0.3	0.614	0.7	7.7	0.3	0.50	7.9	68.6	8	15.8	8

## Supplementary Materials

The following supporting information can be downloaded at: https://www.mdpi.com/article/10.3390/ma15228056/s1, Figure S1: Actual Simulink model diagram of the solar cell; Figure S2: Solar cell block diagram in MATLAB/SIMULINK; Figure S3: Solar cell block parameters in MATLAB/SIMULINK; Figure S4: Simple code for drawing the I-V curve.

## References

[1] Philipps S., Warmuth W. (2022). Photovoltaics Report, Fraunhofer Institute for Solar Energy Systems.

[2] Kim J., Rabelo M., Padi S., Yousuf H., Cho E.-C., Yi J. (2021). A Review of the Degradation of Photovoltaic Modules for Life Expectancy. Energies.

[3] Sabbaghpur Arani M., Hejazi M.A. (2016). The comprehensive study of electrical faults in PV arrays. J. Electr. Comput. Eng..

[4] Katayama N., Osawa S., Matsumoto S., Nakano T., Sugiyama M. (2019). Degradation and fault diagnosis of photovoltaic cells using impedance spectroscopy. Sol. Energy Mater. Sol. Cells.

[5] Hasan A.A.Q. Modeling and Performance Evaluation of Solar Cells Using I-V curve Analysis. Proceedings of the 2nd International Conference on Emerging Technologies and Intelligent Systems (ICETIS 2022).

[6] Livera A., Theristis M., Makrides G., Georghiou G.E. (2018). Recent advances in failure diagnosis techniques based on performance data analysis for grid-connected photovoltaic systems. Renew. Energy.

[7] Zaghba L., Khennane M., Borni A., Fezzani A. (2021). Investigation of the major internal and external factors that affect photovoltaic modules energy production and systems performance. J. Renew. Energy.

[8] Chen Z., Wu L., Cheng S., Lin P., Wu Y., Lin W. (2017). Intelligent fault diagnosis of photovoltaic arrays based on optimized kernel extreme learning machine and I-V characteristics. Appl. Energy.

[9] Alajmi M., Abdel-Qader I. (2016). Fault detection and localization in solar photovoltaic arrays using the current-voltage sensing framework. Proceedings of the 2016 IEEE International Conference on Electro Information Technology (EIT).

[10] Chine W., Mellit A., Lughi V., Malek A., Sulligoi G., Pavan A.M. (2016). A novel fault diagnosis technique for photovoltaic systems based on artificial neural networks. Renew. Energy.

[11] Hasan A.A.Q., Ahmed Alkahtani A., Shahahmadi S.A., Nur EAlam M., Islam M.A., Amin N. (2021). Delamination-and electromigration-related failures in solar panels—A review. Sustainability.

[12] Kumari V., Kumar N., Srivatsa K., Aloysius R., Datta M., Srivastava S.K., Vandana, Rauthan C.M.S., Pathi P. (2020). Estimation of potential induced degradation in solar Mini-modules. Mater. Today Proc..

[13] Finsterle T., Černá L., Hrzina P., Rokusek D., Benda V. (2021). Diagnostics of PID Early Stage in PV Systems. Energies.

[14] Florides M., Makrides G., Georghiou G.E. (2018). Characterisation of the shunt resistance due to potential induced degradation (PID) in crystalline solar cells. Proceedings of the 2018 IEEE 7th World Conference on Photovoltaic Energy Conversion (WCPEC) (A Joint Conference of 45th IEEE PVSC, 28th PVSEC & 34th EU PVSEC).

[15] Luo W., Clement C.E., Khoo Y.S., Wang Y., Khaing A.M., Reindl T., Kumar A., Pravettoni M. (2021). Photovoltaic module failures after 10 years of operation in the tropics. Renew. Energy.

[16] Kwembur I., McCleland J.C., van Dyk E., Vorster F. (2019). Detection of Potential Induced Degradation in mono and multi-crystalline silicon photovoltaic modules. Phys. B Condens. Matter.

[17] Berghold J., Koch S., Böttcher A., Ukar A., Leers M., Grunow P. (2013). Potential-induced degradation (PID) and its correlation with experience in the field. Photovolt. Int..

[18] Hacke P., Terwilliger K., Smith R., Glick S., Pankow J., Kempe M., Bennett S.K.I., Kloos M. (2011). System voltage potential-induced degradation mechanisms in PV modules and methods for test. Proceedings of the 2011 37th IEEE Photovoltaic Specialists Conference.

[19] Spataru S.V., Sera D., Hacke P., Kerekes T., Teodorescu R. (2016). Fault identification in crystalline silicon PV modules by complementary analysis of the light and dark current–voltage characteristics. Prog. Photovolt. Res. Appl..

[20] Sarikh S., Raoufi M., Bennouna A., Benlarabi A., Ikken B. Fault diagnosis in a photovoltaic system through I-V characteristics analysis. Proceedings of the 9th International Renewable Energy Congress (IREC).

[21] Meyer E.L., Van Dyk E.E. (2005). The effect of reduced shunt resistance and shading on photovoltaic module performance. Proceedings of the Conference Record of the Thirty-first IEEE Photovoltaic Specialists Conference, 2005.

[22] Roberts J.J., Zevallos A.A.M., Cassula A.M. (2017). Assessment of photovoltaic performance models for system simulation. Renew. Sustain. Energy Rev..

[23] Somasundaran P., Sinha A., Gupta R. Simulation and characterization of spatial variation of shunts in industrial solar cells by PSpice and dark lock-in infrared thermography. Proceedings of the 27th European Photovoltaic Solar Energy Conference and Exhibition.

[24] Somasundaran P., Gupta R. (2016). Influence of local shunting on the electrical performance in industrial Silicon solar cells. Sol. Energy.

[25] Barbato M., Meneghini M., Giliberto V., Giaffreda D., Magnone P., Derose R., Fiegna C., Meneghesso G. Effect of shunt resistance on the performance of mc-Silicon solar cells: A combined electro-optical and thermal investigation. Proceedings of the 38th IEEE Photovoltaic Specialists Conference.

[26] Voswinckel S., Mikolajick T., Wesselak V. (2020). Influence of the active leakage current pathway on the potential induced degradation of CIGS thin film solar modules. Sol. Energy.

[27] Dong N.C., Islam M.A., Ishikawa Y., Uraoka Y. (2018). The influence of sodium ions decorated micro-cracks on the evolution of potential induced degradation in p-type crystalline silicon solar cells. Sol. Energy.

[28] Dost G., Höffler H., Greulich J.M. (2021). Advanced Series Resistance Imaging for Silicon Solar Cells via Electroluminescence. Phys. Status Solidi.

[29] Bahabry R.R., Hanna A.N., Kutbee A.T., Gumus A., Hussain M.M. (2018). Impact of Nickel Silicide Rear Metallization on the Series Resistance of Crystalline Silicon Solar Cells. Energy Technol..

[30] Bouaichi A., Merrouni A.A., Hajjaj C., Messaoudi C., Ghennioui A., Benlarabi A., Ikken B., El Amrani A., Zitouni H. (2019). In-situ evaluation of the early PV module degradation of various technologies under harsh climatic conditions: The case of Morocco. Renew. Energy.

[31] Chander S., Purohit A., Sharma A., Nehra S., Dhaka M. (2015). Impact of temperature on performance of series and parallel connected mono-crystalline silicon solar cells. Energy Rep..

[32] Goranti S., TamizhMani G. (2012). Potential induced degradation (PID) study on accelerated stress tested PV modules. Proceedings of the 2012 38th IEEE Photovoltaic Specialists Conference.

[33] Hoffmann S., Koehl M. (2012). Effect of humidity and temperature on the potential-induced degradation. Prog. Photovolt. Res. Appl..

