# Supplementary material for: Impact Analysis of Potential Induced Degradation on Crystalline Silicon Solar Cell Performance by Correlating Practical Diagnosis with MATLAB Simulation
Source: Materials (Basel). 2022 Nov 15;15(22):8056. doi: 10.3390/ma15228056 (PMC9699168; doi:10.3390/ma15228056)
Supplement: Supplementary file 1 [file materials-15-08056-s001.zip › materials-1853155-supplementary.pdf]

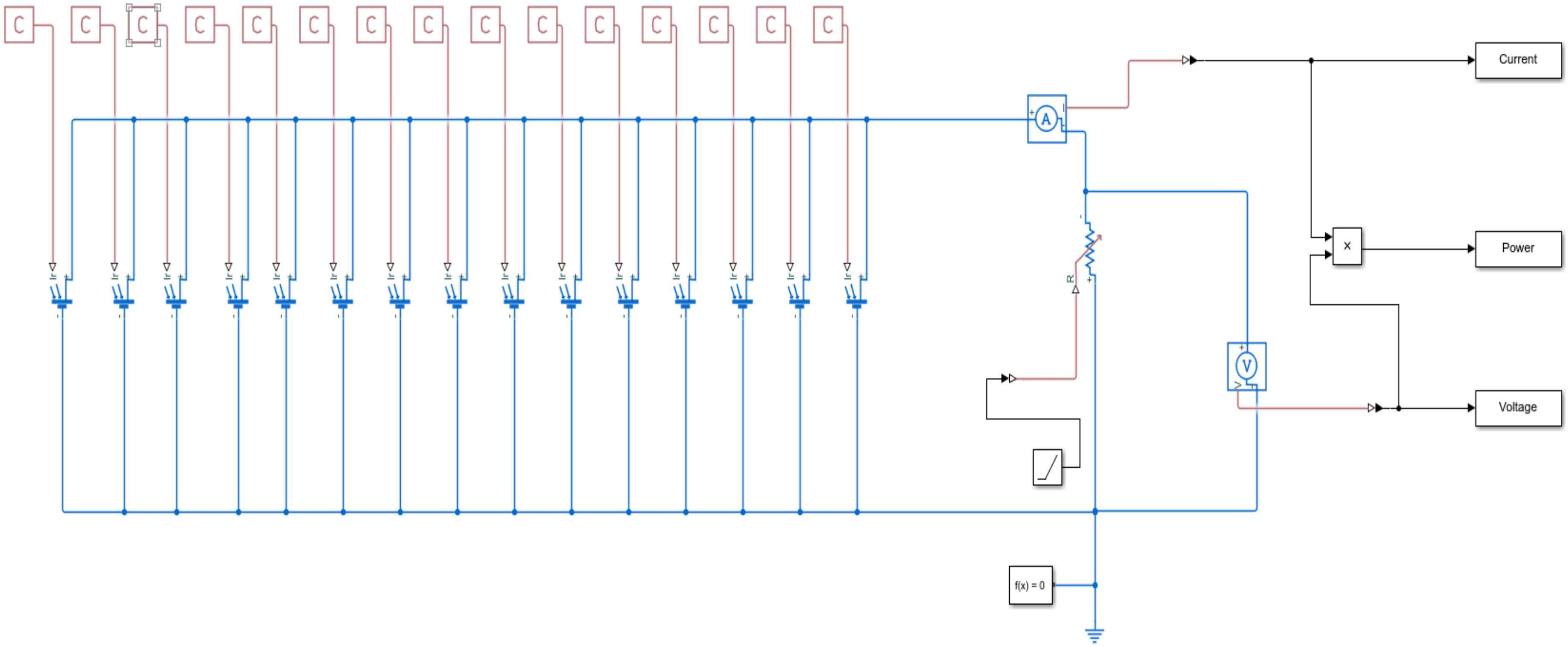

Figure S1. Actual Simulink model diagram of the solar cell.

- **MATLAB/SIMULINK setup**

A mathematical model representing a physical system can be easily expressed and simulated in Simulink. These models are graphically represented as block diagrams. The user can select blocks from the provided libraries to represent various phenomena and models in a number of different formats. As shown in Figure S2 below, one of the models available in the Simulink library is a solar cell. Furthermore, current and voltage sensors are used to read measure the current and voltage under the effect of solar irradiance of (1000 W/m^2). The block diagram of a solar cell is derived from the equivalent circuit Equation 1 presented in the main text of the paper, and the block parameters are depicted in Figure S3. In addition, other block diagrams are used to run the mathematical calculations, such as ramp and solver configuration.

Solar cell model characteristics in Simulink software provide three different block parameterization settings named by s/c current and o/c voltage, five parameters, by equivalent circuit parameters, five parameters, and by equivalent circuit parameters, eight parameters. The first two parameterization settings provide only a few parameters to be adjusted; thus, a parameter such as shunt resistance is unavailable. However, the third parameterization settings allow the user to have complete control of the parameters and, most notably, the series and shunt resistances of the solar cell model.

After connecting the blocks in to create the solar cell model, the data could be extracted directly from workspace in MATLAB main page. As can be seen in Figure S4, the value of voltage and current are displayed as column data. The same Figure is also showing the simple code written to draw the I-V curve.

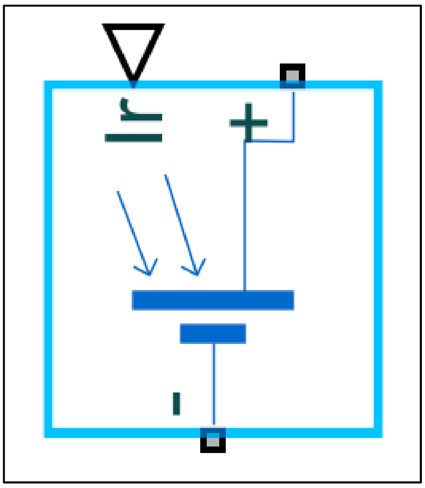

Figure S2. Solar cell block diagram in MATLAB/SIMULINK.

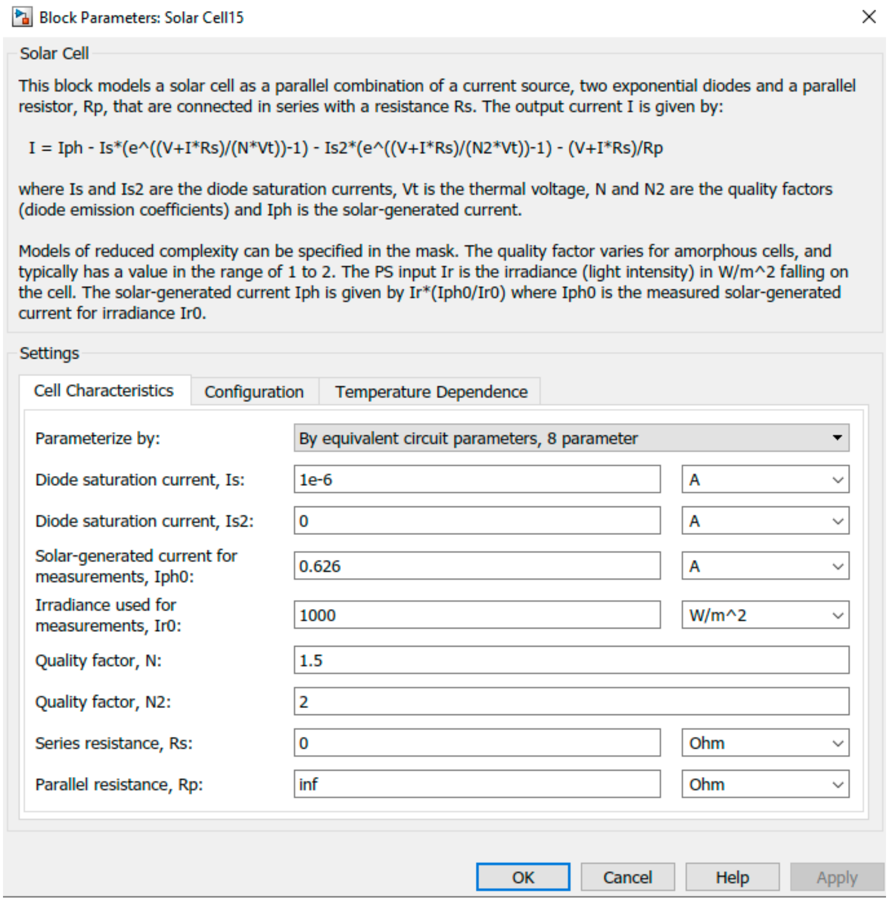

Figure S3. Solar cell block parameters in MATLAB/SIMULINK.

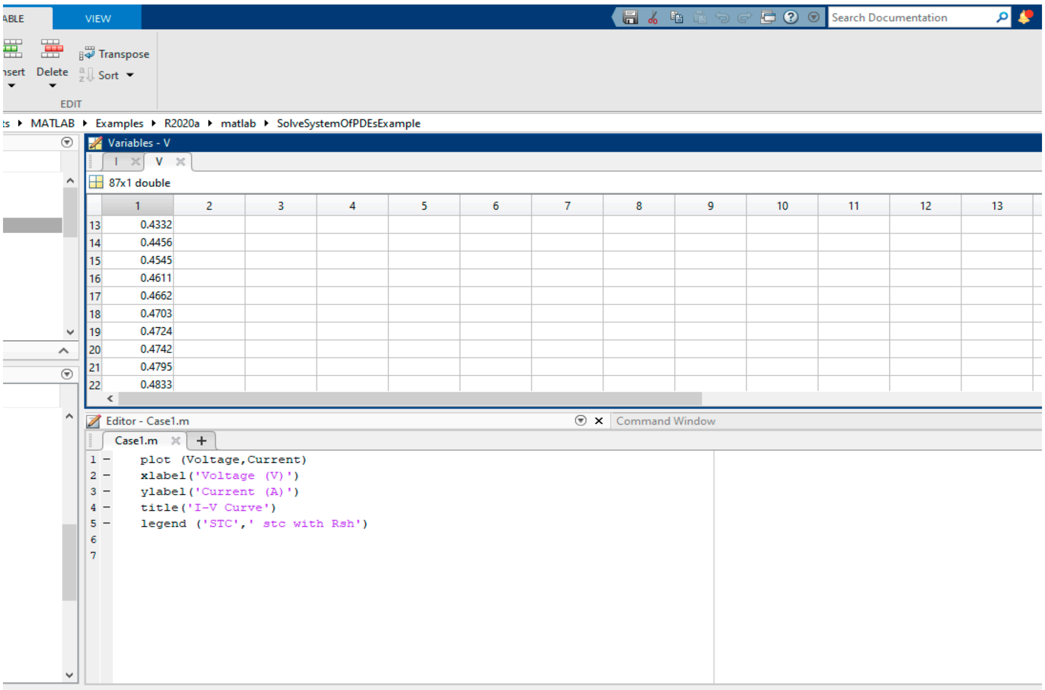

Figure S4. Simple code for drawing the I-V curve.
